# Investigation of the Corrosion Behavior of L245 Steel in 3.5 wt.% NaCl Solution with Varying Concentrations of Na_2_S_2_O_3_

**DOI:** 10.3390/ma18102270

**Published:** 2025-05-14

**Authors:** Mingyu Bao, Yan He, Jing Li, Lingfan Zhang, Chang Liu, Lei Wang, Zidan Wen, Xiaoyan Zhang, Shuliang Wang

**Affiliations:** 1 Safety, Environment & Technology Supervision Research Institute, PetroChina Southwest Oil & Gasfield Company, Chengdu 610041, China; 2 Sichuan Changning Natural Gas Development Co., Ltd., Chengdu 610000, China; 3 School of New Energy and Materials, Southwest Petroleum University, Chengdu 610500, China

**Keywords:** corrosion, L245 steel, electrochemical behaviour, Cl^−^/S_2_O_3_^2−^

## Abstract

In the extraction of shale gas, Cl^−^ and S_2_O_3_^2−^ are one of the important factors causing severe corrosion and failure of equipment and pipelines. Addressing the Cl^−^/S_2_O_3_^2−^ corrosion challenge in shale gas exploitation pipeline steels, this study evaluates the corrosion rates of L245 steels under diverse conditions, including S_2_O_3_^2−^ concentration and exposure time, utilizing the weight loss method. The microstructural, elemental, and phase compositions of the corrosion products were examined, and the electrochemical behavior of L245 steel was scrutinized under various conditions. Findings indicate that S_2_O_3_^2−^ addition intensifies localized corrosion on L245 steel, with the corrosion nature being contingent upon S_2_O_3_^2−^ concentration in the Cl^−^-containing solution. Concurrently, an escalation in S_2_O_3_^2−^ concentration correlates with a reduction in capacitive arc diameter and a significant decrease in film resistance, culminating in an accelerated corrosion rate.

## 1. Introduction

In recent years, many countries have been continuously exploring the extraction of natural gas from shale to ensure energy security, and the development of shale gas extraction has been rapid [1]. In the extraction of shale gas, corrosion is an important factor affecting safety, and failures caused by it account for about 25% of all equipment failures [2], among which Cl^−^ and S_2_O_3_^2−^ are one of the important factors causing severe corrosion and failure of equipment and pipelines. Pipelines are affected by chlorides, sulfides, and formation water, which can lead to severe corrosion [3,4]. Among these corrosive media, polysulfates, especially thiosulfate ions S_2_O_3_^2−^, have been identified as one of the most harmful substances in the oil and gas industry [5,6,7]. S_2_O_3_^2−^ alone does not cause corrosion, but when S_2_O_3_^2−^ and high concentrations of Cl^−^ coexist, it can lead to severe pitting or crevice corrosion [8,9,10,11]. However, when the concentration of S_2_O_3_^2−^ is high, it can mitigate pitting corrosion, as a large amount of S_2_O_3_^2−^ can neutralize the acidic solution in the pit [12,13,14].

Given the severe corrosion phenomena caused by the synergistic effect of S_2_O_3_^2−^ and Cl^−^, the corrosion mechanism of S_2_O_3_^2−^ and Cl^−^ on various metals or alloys has attracted widespread attention [14,15,16,17]. Fu et al. [18] studied the effect of thiosulfate ions on alloy 800 through electrochemical research and found that under the synergistic action of S_2_O_3_^2−^ and Cl^−^, Cl^−^ is first absorbed onto the alloy surface to destroy the passive film, while S_2_O_3_^2−^ adsorbs on the damaged passive film surface and reacts with the alloy matrix, hindering the passivation of alloy 800. Zakeri et al. [19] also found that the pit repassivation potential decreases with the addition of S_2_O_3_^2−^. In the presence of S_2_O_3_^2−^, a significant decrease in the critical concentration of cations within a single corrosion pit may indicate its effect on the repassivation potential by reducing the critical concentration of pit chemistry. Cui et al. [20] demonstrated through potentiostatic current and electrochemical impedance spectroscopy (EIS) that S_2_O_3_^2−^ promotes the dissolution process of the passive film, increases the passive current density, and reduces the polarization resistance. Choudhary et al. [21] discovered that pitting corrosion occurs in S_2_O_3_^2−^ solutions because the thin film on the alloy substrate and the S_2_O_3_^2−^ on the free surface are reduced to adsorbed sulfur during the reaction process, which accelerates the anodic dissolution and inhibits the repassivation process. Zhang et al. [22] demonstrated that under high anodic potentials, S_2_O_3_^2−^ is reduced to H_2_S, and H_2_S catalyzes the anodic dissolution of duplex stainless steel, with the reaction as follows:

(1) $$S_{2}O_{3}^{2-}+6H^{+}+4e^{-}\to 2S+3H_{2}O$$

or disproportionate reaction:

(2) $$S_{2}O_{3}^{2-}+H^{+}=S+HSO_{3}^{-}$$

The generated sulfur combines with H^+^:

(3) $$S+2H^{+}+2e^{-}=H_{2}S$$

H_2_S ultimately reacts with metal ion M:

(4) $$M^{n+}+\frac{n}{2}H_{2}S=MS_{\frac{n}{2}}+nH^{+}$$

Ning et al. [23] confirmed that thiosulfate (S_2_O_3_^2−^) can be reduced to aqueous hydrogen sulfide (H_2_S) within pits at low potentials or under acidic pitting conditions, promoting the active dissolution of metal within the pits.

Although extensive research has been conducted on the synergistic corrosion of nickel-based alloys and stainless steels by S_2_O_3_^2−^ and Cl^−^, there is no mature research on the corrosion problems of L245 steel, which is commonly used in shale gas production and transportation pipelines, in environments containing S_2_O_3_^2−^ and Cl^−^. This article aims to explore the corrosion mechanism and patterns by studying the effects of different concentrations of S_2_O_3_^2−^ solution containing Cl^−^ and different immersion times on L245 steel, providing theoretical support and experimental basis for the corrosion and protection of L245 steel in the field of shale gas.

## 2. Materials and Methods

### 2.1. Sample Preparation

Specimens measuring 40 mm × 13 mm × 2 mm were sectioned from commercially procured L245 steel produced by Xinyou Instrument Factory, Gaoyou, China, with chemical compositions detailed in Table 1. Prior to the weight-loss experiment, specimens were meticulously polished with 150#, 600#, and 800# sandpaper to achieve a smooth, defect-free surface. Subsequent degreasing with acetone, cleaning with alcohol, and drying with cold air preceded final weighing and recording in a desiccated state within a drying oven.

### 2.2. Weight-Loss Experiment

A 3.5 wt.% NaCl solution was prepared using deionized water, to which Na_2_S_2_O_3_ was added at concentrations of 0 mol/L, 0.5 mol/L, 1.0 mol/L, and 1.5 mol/L. To mitigate O_2_ interference with Na_2_S_2_O_3_, the deionized water was purged with high-purity N_2_ for 4 h prior to Na_2_S_2_O_3_ introduction. Experiments were conducted at a controlled temperature of 40 °C for immersion periods of 5, 10, and 15 days. And the corrosion solution was replaced every 7 days. The corrosion samples were retrieved, and the macroscopic morphology of the corrosion products was documented after drying. Surface corrosion products were removed in accordance with ISO 8407:2021 [24], followed by cleaning, drying, and reweighing by a scale with an accuracy of 0.1 mg to calculate the corrosion rate using Formula (5) [25].

(5) $$CR=\frac{87600\Delta w}{\rho St}$$

where *CR* represents the corrosion rate with the unit mm/a, 87,600 is a constant, Δ*w* is the mass loss due to corrosion before and after the experiment with the unit g, *ρ* is the density of the steel (7.85 g/cm^3^) with the unit g/cm^3^, *S* is the surface area of the specimen (12.52 cm^2^) with the unit cm^2^, and *t* is the immersion time with the unit h. To reduce accidental errors, three specimens were prepared for each experiment.

### 2.3. Corrosion Product Analysis

Corrosion products on the corroded L245 steel were analyzed using a DX-2700 X-ray diffractometer manufactured by Haoyuan Instrument Co., Ltd., Dandong, China to determine the phases. The test angle range was 10°~70°, with a step of 0.02° and a scanning speed of 2°/min. The corrosion surface morphology of L245 steel was observed using secondary electron by a ZEISS EV0 MA15 scanning electron microscope (SEM) manufactured by Carl Zeiss Co., Ltd., Oberkochen, Germany, with an accelerating voltage of 20 kV and 50 nm step size. The chemical composition of corrosion products was analyzed by energy-dispersive spectroscopy (EDS) integrated with the SEM. Finally, the product composition was ascertained through a synthesis of SEM-EDS data and X-ray diffraction results.

### 2.4. Electrochemical Testing

The post-immersion specimens were coated with epoxy resin, exposing only a 1 cm^2^ area as the active surface. Electrochemical experiments were performed using a PGSTAT302N workstation manufactured by Metrohm AG Ltd., Herisau, Switzerland, employing a three-electrode configuration with the L245 steel as the working electrode, a platinum plate as the counter electrode, and a saturated calomel electrode as the reference. Open-circuit potential (OCP) was monitored for 2000s to ensure system stabilization. EIS was conducted within a frequency spectrum of 10^5^ to 10^−2^ Hz at an amplitude of ±5 mV. Data were analyzed using ZSimpWin software 3.60, and potentiodynamic polarization curves were generated from −0.5 to 1.0 V (Vs. OCP) at a scan rate of 1 mV/s, with data fitting performed using OriginPro 2024b 10.1.5.132 to ascertain *i*_corr_ and *E*_cor_ values.

## 3. Results

### 3.1. Corrosion Weight Loss Test

**Figure 1:**
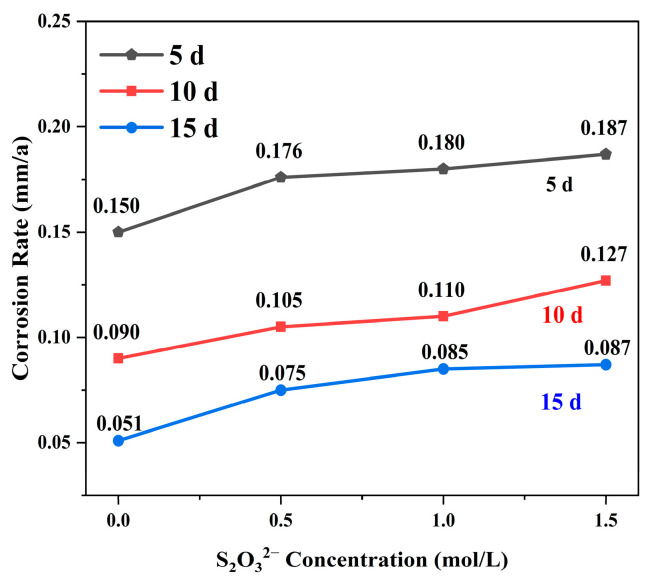
Average corrosion rates of L245 steel in corrosive medium of different concentrations over different time intervals.

Figure 1 delineates the corrosion rates of L245 steel in media of varying concentrations over different time intervals. It is observed that, at equivalent exposure durations, the average corrosion rate escalates with increasing S_2_O_3_^2−^ concentration. Conversely, at constant S_2_O_3_^2−^ concentrations, the corrosion rate diminishes with extended immersion periods, suggesting that surface-forming corrosion products may impede further corrosion [26].

### 3.2. Analysis of the Corrosion Product

#### 3.2.1. Macroscopic Corrosion Morphology

**Figure 2:**
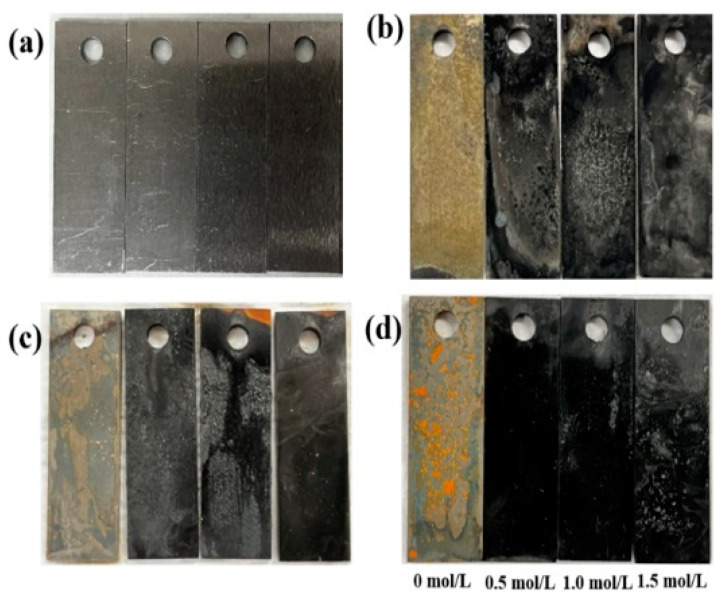
Corrosion morphologies of immersion in solutions with different concentrations (from left to right: 0 mol/L, 0.5 mol/L, 1.0 mol/L, 1.5 mol/L S_2_O_3_^2−^). (**a**) 0 days, (**b**) 5 days, (**c**) 10 days, (**d**) 15 days.

Figure 2 shows the samples corroded for different durations at various concentrations of S_2_O_3_^2−^ in a 3.5 wt.% NaCl solution. As shown in Figure 2, L245 steel shows different morphology after corrosion in solution containing S_2_O_3_^2−^ and without S_2_O_3_^2−^. It is obvious that the steel surface corroded in the solution containing S_2_O_3_^2−^ is covered with a layer of uneven black corrosion products compared with the solution without S_2_O_3_^2−^. In addition, the L245 steels with 1.0 mol/L and 1.5 mol/L S_2_O_3_^2−^ had thick corrosion products on the surface and showed severe localized corrosion, which shows that the increase in S_2_O_3_^2−^ concentration can promote the localized corrosion and the formation of corrosion products. Moreover, localized corrosion occurred under the combined action of Cl^−^ and S_2_O_3_^2−^, which is consistent with the conclusions obtained by Ning et al. [23].

#### 3.2.2. Phase Analysis by XRD

**Figure 3:**
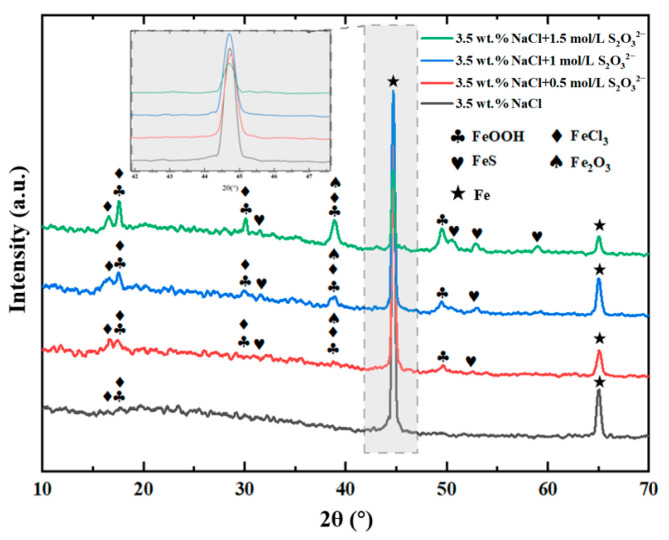
XRD patterns of L245 steel after corrosion for 15 days under four different conditions.

Figure 3 shows the XRD spectra of L245 steel after being corroded for 15 days under four different conditions. The XRD patterns reveal the presence of different phases on the corrosion film of L245 steel in NaCl solutions with varying concentrations of S_2_O_3_^2−^, including FeOOH, FeCl_3_, FeS, and Fe_2_O_3_. In the solutions without S_2_O_3_^2−^, the corrosion films are composed of FeOOH, FeCl_3_, and Fe_2_O_3_. Although the dissolution of steel in NaCl solution first produces Fe^2+^, it is more common for these products to be oxidized to Fe^3+^ when exposed to the air [27]. The presence of FeS peaks in the XRD patterns of the Cl^−^/S_2_O_3_^2−^ solution clearly describes the formation of an FeS layer on the surface of the carbon steel.

#### 3.2.3. Corrosion Product and Elemental Composition Analysis by SEM-EDS

**Figure 4:**
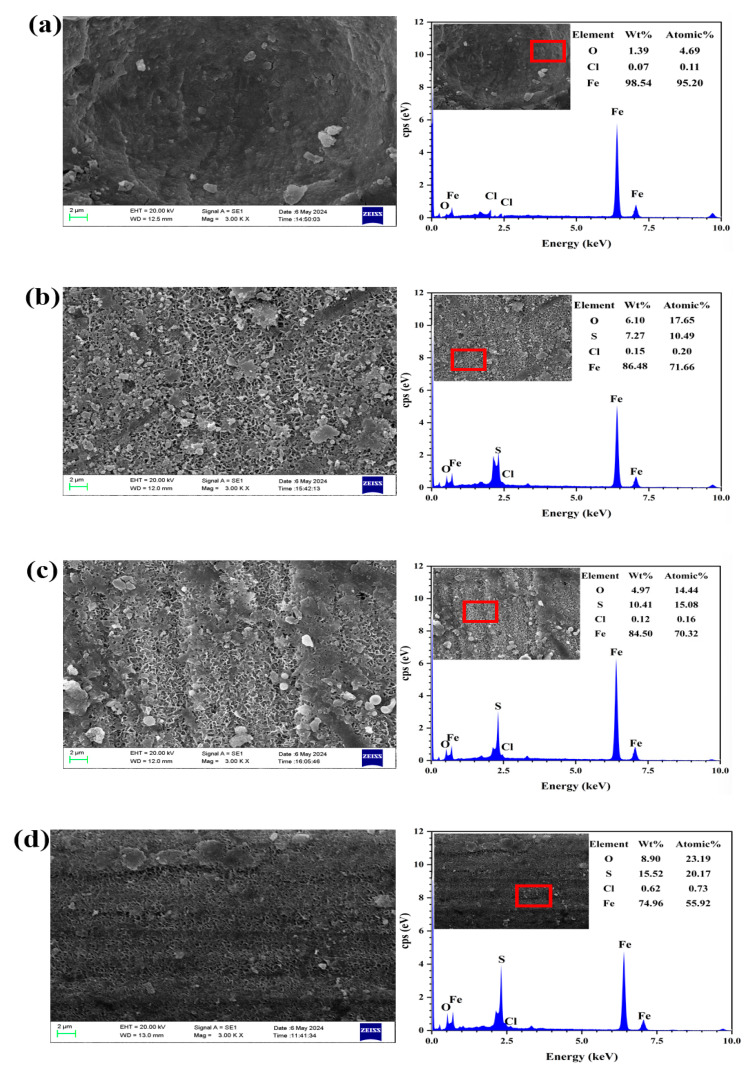
SEM SE surface morphology and energy spectrum of L245 steel samples after being corroded for 15 days in a 3.5 wt.% NaCl solution with varying concentrations of S_2_O_3_^2−^: (**a**) 0 mol/L, (**b**) 0.5 mol/L, (**c**) 1.0 mol/L, (**d**) 1.5 mol/L.

Figure 4 shows the surface morphology and energy spectrum of L245 steel samples after being corroded for 15 days in a 3.5 wt.% NaCl solution with different concentrations of S_2_O_3_^2−^. The corrosion morphologies of L245 steels at different S_2_O_3_^2−^ concentrations also showed differences. As can be seen from Figure 4a, a thick layer of corrosion products has formed on the surface of the corroded L245 steel, which is relatively dense and has a certain protective effect on the substrate, but it cannot effectively prevent the corrosive medium from entering the film/substrate interface to corrode the substrate. In the pure NaCl solution, the main elements of the corrosion products are C, O, Fe, and a small amount of Cl, which indicates that the corrosion film is mainly composed of iron oxides and chlorides, such as FeOOH and Fe_2_O_3_ [28,29].

From Figure 4b, it can be seen that a thick layer of corrosion products has formed on the surface of the corroded L245 steel with S_2_O_3_^2−^, which is loose and has many pores, providing poor protection to the substrate and failing to effectively prevent the corrosive medium from entering the film/substrate interface to corrode the substrate. Compared with Figure 4a, the corrosion is more severe. The main elements of corrosion products are C, O, Fe, S, and a small amount of Cl, indicating that the corrosion film is mainly composed of iron sulfides, oxides, and chlorides. The S content at the corroded site is 7.18 wt.%, indicating that iron sulfides dominate in the intact corrosion products. However, when the concentration of S_2_O_3_^2−^ is low, it is difficult to form a dense, protective corrosion product film, and no dense iron sulfide corrosion products are observed on the sample surface.

From Figure 4c, it can be seen that a thick layer of corrosion products has formed on the surface of L245 after corrosion, which is loose and has many evenly distributed larger pores, providing poor protection to the substrate. The main corrosion products are C, O, Fe, S, and a small amount of Cl, among which the S content reaches 10.41 wt.%, indicating that the corrosion products are mainly composed of iron sulfides, with some oxides and a small amount of chlorides.

Analyzing Figure 4d, it can be found that compared with Figure 4b,c, the surface of L245 steel has produced a denser corrosion product, and the diameter of the pores has also been relatively reduced, indicating that the increase in S_2_O_3_^2−^ concentration has intensified the corrosion process, resulting in a denser corrosion product film. The elements of the corrosion products are also mainly Fe, O, Cl, and S, with the S content in the elements reaching as high as 20.17 wt.%, which is the highest S content among the four states. This indicates that as the concentration of S_2_O_3_^2−^ increases, the proportion of S element in the corrosion products also increases. In addition, under this condition, the high concentration of S_2_O_3_^2−^ is conducive to the precipitation of Fe sulfides; hence, the surface is observed to have denser and thicker corrosion products. The research of Cao et al. [30] also proved this point.

**Figure 5:**
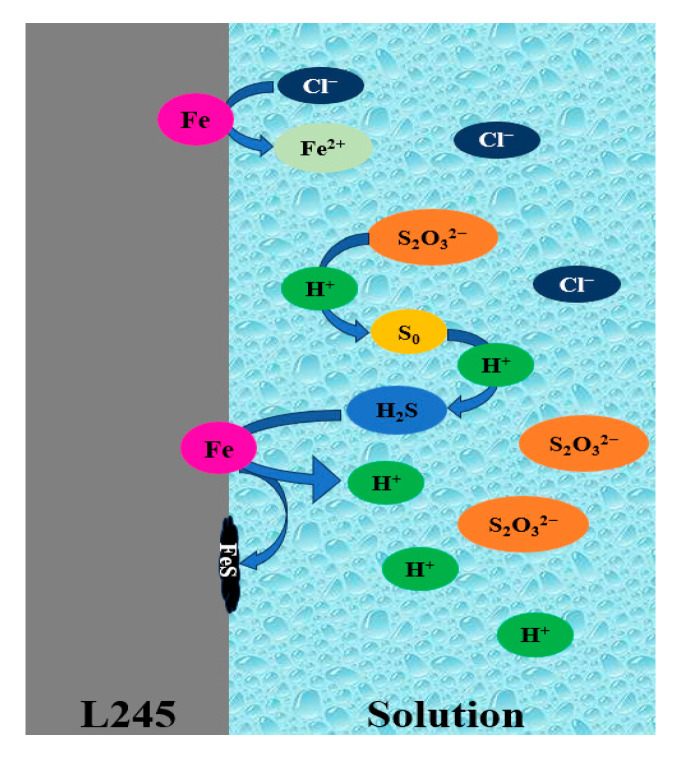
The schematic diagram of Cl^−^/S_2_O_3_^2−^ corrosion.

L245 steel exhibits uniform corrosion in pure NaCl solution, while obvious local corrosion is observed when Cl^−^ and S_2_O_3_^2−^ ions coexist in the solution. EDS experiments showed the elemental analysis results of the products formed on the surface of L245 steel in 3.5 wt.% NaCl solution with different concentrations of S_2_O_3_^2−^: the main components of the corrosion product film in pure NaCl solution are iron oxides and chlorides. When Na_2_S_2_O_3_ is added to the NaCl solution, a sulfur peak appears, indicating the formation of a sulfide layer on the surface. Therefore, the corrosion products are a mixture of iron oxides and chlorides in pure Cl^−^ solution and oxides, chlorides, and sulfides in Cl^−^/S_2_O_3_^2−^ solution. Combined with the relevant literature and XRD results, reactions that produce FeOOH, FeCl_3_, Fe_2_O_3_, and FeS occur during the corrosion process, forming a pseudo-passivation layer [18], which then leads to pitting corrosion. The schematic diagram of Cl^−^/S_2_O_3_^2−^ corrosion is shown in Figure 5. S_2_O_3_^2−^ in the solution reacts with H^+^ to form elemental sulfur, and then reacts with free H^+^ to form H_2_S. H_2_S contacts with the metal matrix and then forms FeS corrosion products, which are covered on the surface of the matrix. Cl^−^ reacts directly with the metal matrix to form FeCl_2_.

### 3.3. Corrosion Electrochemical Testing

#### 3.3.1. Potentiodynamic Polarization Curve

**Figure 6:**
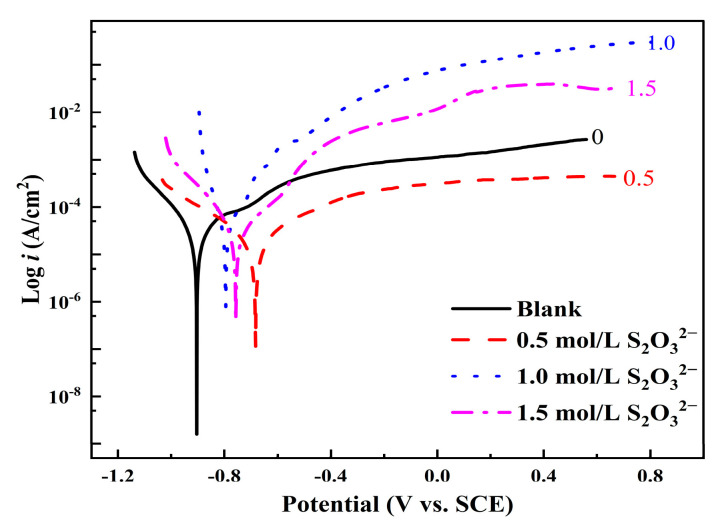
Polarization curves of L245 steel after 5 days of corrosion in 3.5 wt. % NaCl solution with different concentrations of S_2_O_3_^2−^.

Figure 6 shows the polarization curves of L245 steel after 5 days of corrosion in 3.5 wt.% NaCl solution with different concentrations of S_2_O_3_^2−^. It can be observed that both the anodic and cathodic processes of L245 steel are electrochemically controlled, and the polarization curves exhibit nonlinear behavior, which is more pronounced at a concentration of 1.5 mol/L S_2_O_3_^2−^. The solution with 1.5 mol/L S_2_O_3_^2−^ shows a constant current in the potential range of −0.62 V to −0.55 V, indicating the formation of a pseudo-passivation layer on the surface of carbon steel (with a current roughly maintained at 0.12 mA/cm^2^). From the increase in current, it can be seen that this layer is eventually pierced when the breakdown potential (Eb) is −0.55 V. The pseudo-passivation layer is composed of a mixture of sulfides and oxides, but its main component is likely to be sulfides, as the formation of sulfides is thermodynamically more feasible than the formation of oxides [21].

**Figure 7:**
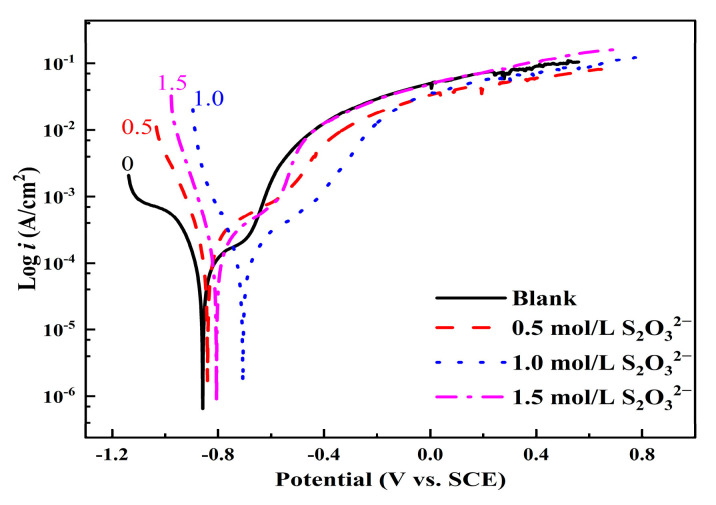
Polarization curves of L245 steel after 10 days of corrosion in 3.5 wt.% NaCl solution with different concentrations of S_2_O_3_^2−^.

Figure 7 shows the polarization curves of L245 steel after 10 days of corrosion in 3.5 wt.% NaCl solution with varying concentrations of S_2_O_3_^2−^. It can be observed that the anodic process of L245 steel is more significantly inhibited under the corrosion conditions of 1.0 mol/L and 1.5 mol/L S_2_O_3_^2−^ concentrations. A region similar to the passivation zone appears during the anodic process. However, judging from the anodic current density in this “passivation zone”, it is not a true passivation zone, as the anodic current in a typical passivation process is generally in the range of a few to several tens of µA/cm^2^. It is obvious that the anodic process is suppressed by the corrosion product film, leading to a decrease in anodic current density. Concurrently, as the concentration of S_2_O_3_^2−^ increases, there is a noticeable fluctuation in the current density near the corrosion potential.

**Figure 8:**
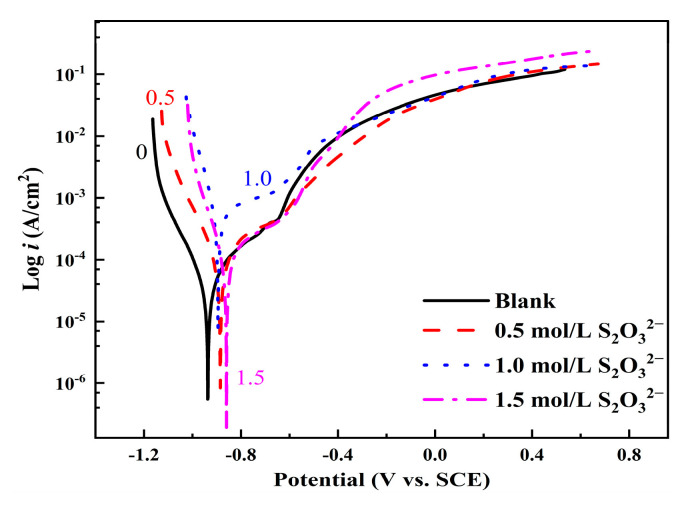
Polarization curves of L245 steel after 15 days of corrosion in 3.5 wt.% NaCl solution with different concentrations of S_2_O_3_^2−^.

Figure 8 shows the polarization curves of L245 steel after 15 days of corrosion in 3.5 wt.% NaCl solution with different concentrations of S_2_O_3_^2−^. It can be observed that the anodic process of L245 steel is also significantly inhibited under the corrosion conditions at a concentration of 0.5 mol/L S_2_O_3_^2−^.

**Figure 9:**
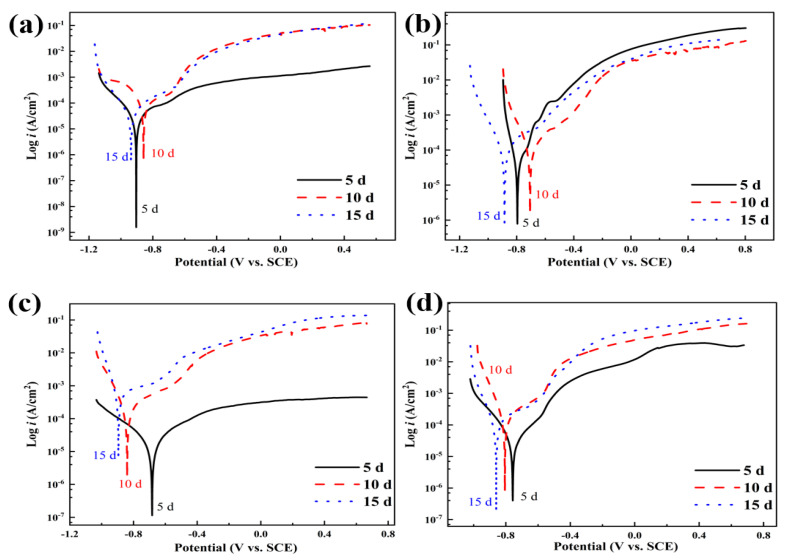
Polarization curves of L245 steel after different durations of corrosion in 3.5 wt.% NaCl solution with varying concentrations of S_2_O_3_^2−^: (**a**) 0 mol/L, (**b**) 0.5 mol/L, (**c**) 1.0 mol/L, (**d**) 1.5 mol/L.

Figure 9 illustrates the polarization curves of L245 steel in solutions with varying concentrations of S_2_O_3_^2−^ and 3.5 wt.% NaCl after being corroded for 5 days, 10 days, and 15 days, respectively. Regardless of the concentration of the corrosive medium, the general trend is that as the number of corrosion days increases, the corrosion product film gradually becomes more complete, significantly suppressing the anodic process. However, due to the composition of the corrosion product film, it fails to protect the substrate, resulting in an increase in corrosion current density and accelerated corrosion rate, which is most evident in solutions with added S_2_O_3_^2−^ and a certain shift in the corrosion potential.

To understand the behavior observed in the presence of Na_2_S_2_O_3_, whether it is due to the sole presence of Cl^−^ ions or the combined effect of S_2_O_3_^2−^ and Cl^−^ ions, polarization experiments were conducted in pure NaCl solution, with results as shown in Figure 9a. With the increase in corrosion days, the polarization behavior does not exhibit the nonlinear behavior observed with the addition of Na_2_S_2_O_3_. At the same time, the corrosion current density increases from 7.9 × 10^−6^ to 3.9 × 10^−5^ A/cm^2^, but it is always lower than that in the presence of Na_2_S_2_O_3_ for the same corrosion days. This indicates that the addition of S_2_O_3_^2−^ promotes corrosion on the surface of carbon steel. However, in the presence of S_2_O_3_^2−^, the curve exhibits nonlinear behavior, which may be due to the formation of a porous and non-protective sulfide layer on the surface. This suggests that the susceptibility to pitting corrosion is enhanced only when both Cl^−^ and S_2_O_3_^2−^ are present. Therefore, when the solution contains only NaCl, carbon steel undergoes uniform corrosion, and when the solution contains both substances, carbon steel may exhibit pitting corrosion, depending on the concentration of these two substances. Wu et al. [31] studied the effects of chloride-to-thiosulfate concentration ratio (CTCR) on the corrosion behavior of passive films in neutral solutions, and confirmed that S_2_O_3_^2−^ has a significant promoting effect on pitting corrosion at high concentration ratios.

The logarithm of current density increases linearly with the applied potential in the range of tens to hundreds of millivolts relative to *E*_corr_. Therefore, the Tafel extrapolation method [32] is used to fit the polarization curve. Table 2 lists the electrochemical parameters such as corrosion current density (*i*_corr_), corrosion potential (*E*_corr_), and Tafel slopes obtained from the fitting of polarization curves under different corrosion conditions using the software built into the system. The polarization curves clearly describe the significant impact of S_2_O_3_^2−^ on the electrochemical behavior of carbon steel in the NaCl solution. A main observation from these polarization curves is that when the concentration of S_2_O_3_^2−^ increases from 0 mol/L to 1.5 mol/L, the icorr value for L245 steel samples corroded for 5 days increases from 7.9 × 10^−6^ A/cm^2^ to 2.7 × 10^−5^ A/cm^2^, and similar increasing trends are observed for samples corroded for 10 days and 15 days. This indicates that the addition of S_2_O_3_^2−^ in the NaCl solution accelerates the corrosion rate of carbon steel.

Due to the presence of S_2_O_3_^2−^ ions in the NaCl solution, the cathodic corrosion current density increases significantly, which is attributed to the reduction reactions of these substances. The typical cathodic reactions that occur on the metal surface are determined by the pH of the solution, the electrochemical potential, and the properties of the corroded surface (passivated/unpassivated). Additionally, the elemental sulfur produced may further react with Fe^2+^ to form an FeS film, thereby accelerating the corrosion process; that is, the addition of S_2_O_3_^2−^ affects both anodic and cathodic reactions in the NaCl solution [33].

The reduction of S_2_O_3_^2−^ to sulfur is more likely to occur on the unpassivated surface of L245 steel. In the NaCl solution, the surface of the L245 steel is not fully covered by an oxide film, which triggers the formation of sulfur, leading to the formation of an FeS layer on the surface of L245 steel. As the concentration of S_2_O_3_^2−^ increases, the formation rate of FeS may increase, resulting in the formation of a pseudo-passivation layer, which can be seen from the constant current region of the polarization curve. At higher anodic potentials, the pseudo-passivation layer breaks down, indicating that carbon steel is susceptible to localized corrosion [34], which is consistent with the surface morphology observations of the corroded samples mentioned earlier. Generally speaking, compared with the oxide film, the sulfide film formed in the early stage of corrosion is more porous and loose and does not provide protection [35]. Therefore, this pseudo-passivation layer cannot fully protect the metal substrate, and uniform corrosion can also occur simultaneously at higher concentrations of S_2_O_3_^2−^.

**Figure 10:**
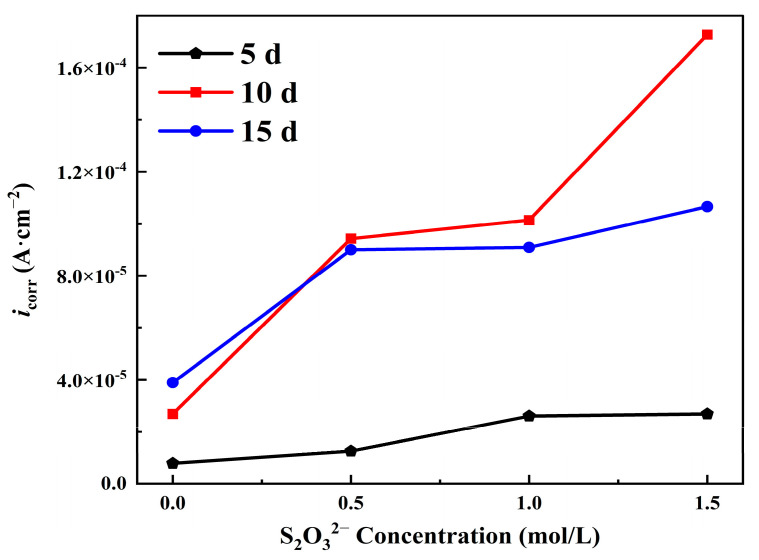
The trend of *i*_corr_ changing with the concentration of S_2_O_3_^2−^.

**Figure 11:**
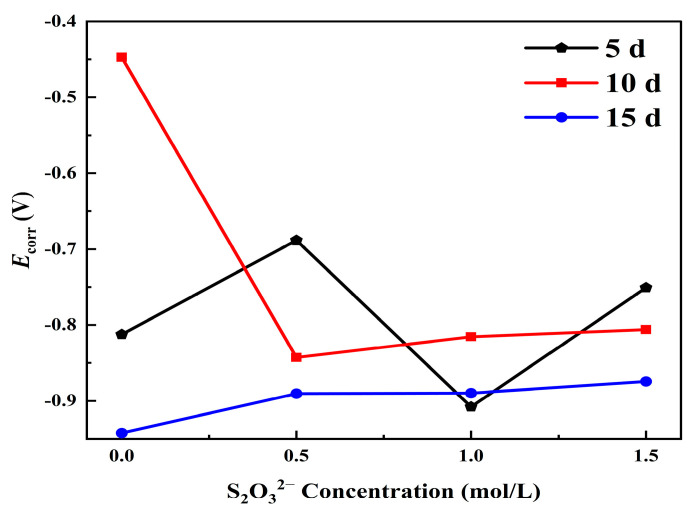
The trend of *E*_cor_ changing with the concentration of S_2_O_3_^2−^.

By plotting the data obtained from the fitting in Table 2, the trends of *E*_corr_ and *i*_corr_ with the change of S_2_O_3_^2−^ concentration can be obtained, as shown in Figure 10 and Figure 11. Comparing Figure 10 with Figure 4, it can be seen that although there are some deviations in the fitting results, the trend of change in corrosion current density of the polarization curve is consistent with the trend of average corrosion rate obtained by the weight loss method mentioned earlier. And the *i*_corr_ increased with the increase in S_2_O_3_^2−^ concentration, which is consistent with the research results of Al-mamun et al. [9] and Kappes et al. [7].

*i*_corr_ increases with the concentration of S_2_O_3_^2−^, which can be explained as follows [7,9,36,37]: In pure NaCl solutions, the passive film formed on the surface of L245 steel has a certain hindrance to Cl^−^, which makes the *i*_corr_ low. When S_2_O_3_^2−^ was added to the solutions containing Cl^−^, the synergistic effect of S_2_O_3_^2−^ and Cl^−^ could promote the degradation of the passive film on the surface of L245 steel, thereby increasing *i*_corr_. In addition, the element S spontaneously generated by the reduction or disproportionation of S_2_O_3_^2−^ also reacts with Fe, resulting in an increase in *i*_corr_. Hence, the addition of S_2_O_3_^2−^ has a significant impact on the corrosion of L245 steel in Cl^−^-containing solutions.

#### 3.3.2. Electrochemical Impedance Spectroscopy Test

**Figure 12:**
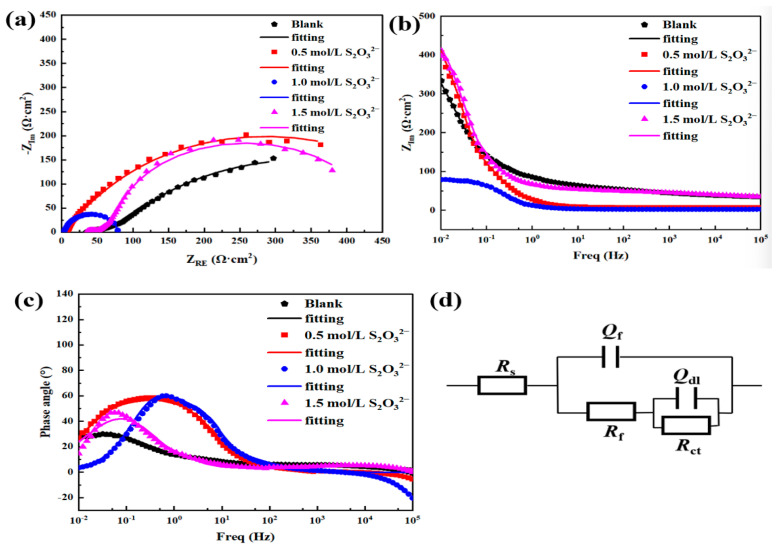
The EIS data obtained for L245 steel after being corroded in 3.5 wt.% NaCl solution with different concentrations of S_2_O_3_^2−^ for 5 days. (**a**) Nyquist plot, (**b**) Bode modulus plot, (**c**) Bode phase angle plot, (**d**) equivalent electric circuit diagram.

Figure 12 presents the EIS data obtained for L245 steel after corrosion under four different concentrations of S_2_O_3_^2−^ at condition 1 (40 °C, 3.5 wt.% NaCl solution, static, 5 days). From the Nyquist plot in Figure 12a, it is evident that upon the addition of S_2_O_3_^2−^, as the concentration of S_2_O_3_^2−^ increases, the diameter of the capacitive arc gradually decreases, and the corresponding film resistance significantly diminishes, leading to an enhanced corrosion rate. This indicates that the higher the concentration of S_2_O_3_^2−^ added, the more severe the corrosion of L245 steel. These findings are in agreement with the conclusions drawn from the previous weight loss tests and polarization measurements.

In order to gain a more in-depth understanding of the corrosion of L245 steel surfaces with added Na_2_S_2_O_3_, the original impedance spectrum data are fitted to an equivalent electric circuit (EEC). Figure 12d represents the equivalent electric circuit model [38,39] corresponding to the EIS data, and taking into account the inhomogeneity of the corrosion product film and the surface of the steel, two constant phase angle elements are used instead of a capacitor, where *R*_s_ denotes the solution resistance, *R*_f_ and *Q*_f_ represent the resistance and capacitance of the passive film, *R*_ct_ and *Q*_dl_ are the charge transfer resistance and the double-layer capacitance, respectively. The fitting parameters are provided in Table 3.

It can be seen that *Q*_f_ decreases with the increase in S_2_O_3_^2−^ concentration after the addition, indicating the thickening of the surface corrosion product film with the increase in S_2_O_3_^2−^ concentration, while the decrease in *n*_f_ indicates the increase in the inhomogeneity of the corrosion product film. In addition, *R*_ct_ decreasing with increasing S_2_O_3_^2−^ concentration shows that the charge transfer process is accelerated and the corrosion rate increased, which is consistent with the results of the polarization curves.

**Figure 13:**
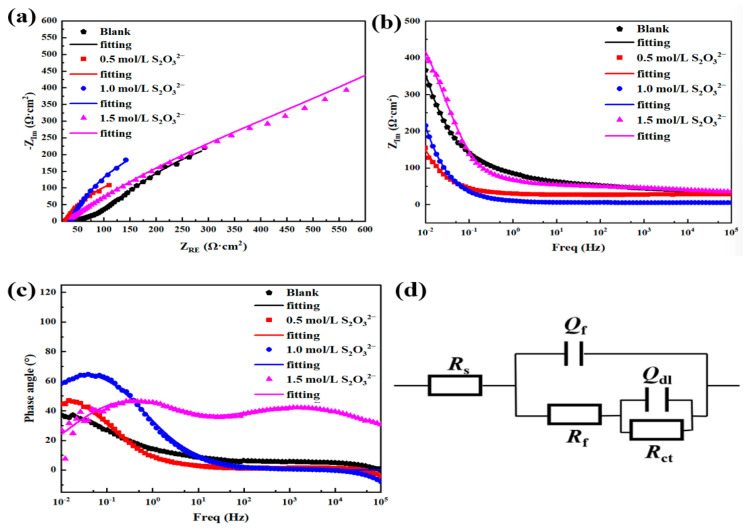
EIS data of L245 steel corroded for 10 days in 3.5 wt.% NaCl solution with different concentrations of S_2_O_3_^2−^. (**a**) Nyquist plot, (**b**) Bode modulus plot, (**c**) Bode phase angle plot, (**d**) equivalent electric circuit diagram.

Figure 13 presents the EIS data obtained for L245 steel after corrosion under four different concentrations of S_2_O_3_^2−^ at condition 2 (40 °C, 3.5 wt.% NaCl solution, static, 10 days). From the Nyquist plot in Figure 13a, it can be observed that as the concentration of S_2_O_3_^2−^ increases, the diameter of the capacitive arc decreases, and the corresponding film resistance significantly decreases, leading to an increased corrosion rate. The trend in EIS analysis with a corrosion period of 10 days is consistent with that of 5 days, and the results are also in agreement with the corrosion rate variation laws determined by weight-loss experiments. At the same time, it can be seen from Figure 13a that in all cases, two time constants (capacitive loops) are observed in the impedance spectra after 10 days of corrosion.

From Table 4, it is evident that as the concentration of S_2_O_3_^2−^ increases, *R*_f_ decreases. Consequently, the total impedance associated with the Faradaic reaction decreases with the increasing concentration of S_2_O_3_^2−^. Additionally, with the increase in S_2_O_3_^2−^ concentration, *R*_ct_ decreases. The results indicate that the rate of the cathodic reaction increases as the concentration of S_2_O_3_^2−^ increases. The total impedance caused by these parameters decreases with the increase in S_2_O_3_^2−^ concentration. This corresponds to the increased corrosion rate with the increase in S_2_O_3_^2−^ concentration observed in the polarization measurements mentioned earlier.

**Figure 14:**
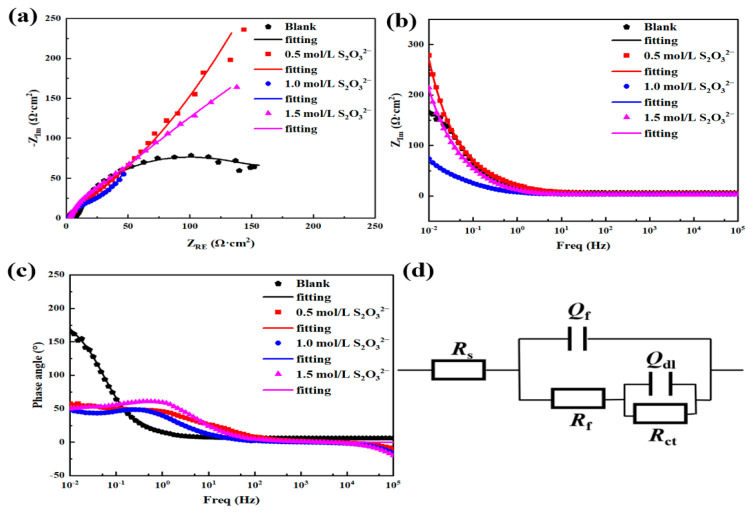
EIS data of L245 steel corroded for 15 days in 3.5 wt.% NaCl solution with different concentrations of S_2_O_3_^2−^. (**a**) Nyquist plot, (**b**) Bode modulus plot, (**c**) Bode phase angle plot, (**d**) equivalent electric circuit diagram.

Figure 14 shows the impedance spectra obtained for L245 steel after corrosion under four different concentrations of S_2_O_3_^2−^ at condition 3 (40 °C, 3.5 wt.% NaCl solution, static, 15 days). It can be observed that two time constants (capacitive loops) are also seen in the impedance spectra after 15 days of corrosion in the presence of S_2_O_3_^2−^. Therefore, the equivalent electrical circuit shown in Figure 14d was used to quantitatively analyze the impedance data. From the Nyquist plot in Figure 14a, it can be seen that as the concentration of S_2_O_3_^2−^ increases, the diameter of the capacitive arc decreases, and the corresponding film resistance significantly decreases, leading to an increased corrosion rate. The results are consistent with the changes in corrosion rate obtained from weight loss and polarization tests. Table 5 provides the corresponding EIS fitting results.

It can be seen that after 15 days of immersion, *Q*_f_ increases with increasing S_2_O_3_^2−^ concentration, indicating a decrease in the thickness of the corrosion product film, while the decrease in *n*_f_ indicates an increase in the inhomogeneity of the corrosion product film. The thinness and inhomogeneity of the corrosion product film promote the reaction between the corrosion medium and the collective, and thus the corrosion rate is increased. Moreover, the decrease in *R*_f_ and *R*_ct_ also shows an increase in corrosion rate.

As expected from the polarization behavior, uniform corrosion was observed on the surface of L245 steel in the pure NaCl solution, while localized corrosion was observed in the presence of S_2_O_3_^2−^, and the phenomenon of localized corrosion became more pronounced with increasing concentration of S_2_O_3_^2−^. This phenomenon supports the findings from weight-loss experiments and polarization measurements, which suggest that when Cl^−^ and S_2_O_3_^2−^ ions are present in the solution simultaneously, a pseudo-passivation layer is formed, which is further eroded by Cl^−^ ions, leading to localized corrosion with a tendency towards pitting.

## 4. Conclusions

The corrosion of L245 steel commonly used in shale gas gathering and transmission pipelines in neutral environments containing S_2_O_3_^2−^ and Cl^−^ has been studied. The main conclusions are as follows:

The results of weight-loss experiments and potentiodynamic polarization tests confirmed that S_2_O_3_^2−^ had a significant effect on the corrosion of L245 steels in Cl^−^-containing solutions. The synergistic effect of S_2_O_3_^2−^ and Cl^−^ could promote the localized corrosion of L245 steels, and the corrosion rate of L245 steel increases with the increase in S_2_O_3_^2−^ concentration. Moreover, under the same corrosive medium conditions, the longer the corrosion time, the smaller the corrosion rate.

S_2_O_3_^2−^ has an obvious effect on the formation of surface corrosion products of L245 steel. The higher the concentration of S_2_O_3_^2−^, the thicker the films formed on the surface of L245 steels. However, the corrosion product films are porous and loose, offering very weak protection. EDS and XRD results indicate that the main corrosion products in this experiment are FeCl_3_, Fe_2_O_3_, and FeOOH, and the addition of S_2_O_3_^2−^ leads to the formation of FeS in addition to Fe oxides and chlorides.

The concentration of S_2_O_3_^2−^ can affect the surface corrosion morphology of L245 steel. The addition of S_2_O_3_^2−^ causes localized corrosion on the surface of L245 steel, and the nature of corrosion (uniform or localized) depends on the CTCR. Uniform corrosion occurs when S_2_O_3_^2−^ is not added, while localized corrosion is observed when S_2_O_3_^2−^ is added. Therefore, referring to this study, the corrosion protection of L245 steel in shale gas exploitation could be possible by regulating CTCR.

This article only studied the ideal corrosion situation of S_2_O_3_^2−^ containing Cl^−^, while in reality, formation water may also contain other ions such as SO_4_^2−^ and CO_3_^2−^ that can affect corrosion. Therefore, the study of the effects of other ions or the combined action of multiple ions on S_2_O_3_^2−^ corrosion should have significant practical implications.

## Figures and Tables

**Table 1:** Chemical composition of L245 steel (wt.%).

Element	C	Si	Mn	P	S	Cr	Ni	Mo	V	Nb	Ti	Fe
L245	0.202	0.254	0.396	0.0189	0.0139	0.023	0.020	0.018	0.0014	0.003	0.0025	Bal.
API 5L	≤0.26	-	≤1.20	≤0.030	≤0.030	-	-	-	Nb + V ≤ 0.06; Nb + V+Ti ≤ 0.15			

**Table 2:** Corrosion potential (*E*_corr_) and corrosion current density (*i*_corr_) values obtained from polarization curves.

Days	Corrosion Medium Concentration	*Β*_a_ (mV/dec)	−*β*_c_ (mV/dec)	*E*_corr_ (V)	*I*_corr_ (A/cm^2^)
5 d	3.5 wt.%NaCl	257	146	−0.81	7.8 × 10^−6^
	3.5 wt.% NaCl + 0.5 mol/L S_2_O_3_^2−^	213	186	−0.69	1.3 × 10^−5^
	3.5 wt.% NaCl + 1.0 mol/L S_2_O_3_^2−^	73	29	−0.91	2.6 × 10^−5^
	3.5 wt.% NaCl + 1.5 mol/L S_2_O_3_^2−^	192	139	−0.75	2.7 × 10^−5^
10 d	3.5 wt.% NaCl	359	170	−0.45	2.7 × 10^−5^
	3.5 wt.% NaCl + 0.5 mol/L S_2_O_3_^2−^	291	104	−0.84	9.4 × 10^−5^
	3.5 wt.% NaCl + 1 mol/L S_2_O_3_^2−^	205	126	−0.82	1.0 × 10^−4^
	3.5 wt.% NaCl + 1.5 mol/L S_2_O_3_^2−^	196	30	−0.81	1.7 × 10^−4^
15 d	3.5 wt.% NaCl	213	127	−0.94	3.9 × 10^−5^
	3.5 wt.% NaCl + 0.5 mol/L S_2_O_3_^2−^	250	120	−0.89	9.0 × 10^−5^
	3.5 wt.% NaCl + 1 mol/L S_2_O_3_^2−^	250	69	−0.89	9.1 × 10^−5^
	3.5 wt.% NaCl + 1.5 mol/L S_2_O_3_^2−^	335	81	−0.87	1.1 × 10^−4^

**Table 3:** Optimal EEC parameters obtained by dissolution of L245 in different corrosive medium (5 d).

EEC Parameters	3.5 wt.% NaCl	3.5 wt.% NaCl + 0.5 mol/L S_2_O_3_^2−^	3.5 wt.% NaCl + 1 mol/L S_2_O_3_^2−^	3.5 wt.% NaCl + 1.5 mol/L S_2_O_3_^2−^
*R*_s_ (Ωcm^−2^)	33.00	7.15	2.76	33.26
*Q*_f_ (Ω^−1^s^n^cm^−2^)	3.141 × 10^−3^	9.683 × 10^−3^	7.092 × 10^−3^	1.021 × 10^−3^
*n*_f_	0.3400	0.8046	0.7649	0.4319
*R*_f_ (Ωcm^−2^)	48.87	29.26	27.33	24.04
*Q*_dl_ (Ω^−1^s^n^cm^−2^)	9.350 × 10^−3^	8.866 × 10^−3^	8.088 × 10^−3^	1.095 × 10^−2^
*n*_dl_	0.6047	0.8841	0.8574	0.8392
*R*_ct_ (Ωcm^−2^)	1046	527	438	283

**Table 4:** Optimal EEC parameters obtained from the dissolution of L245 in different corrosive media (10 d).

EEC Parameters	3.5 wt.% NaCl	3.5 wt.% NaCl + 0.5 mol/L S_2_O_3_^2−^	3.5 wt.% NaCl + 1 mol/L S_2_O_3_^2−^	3.5 wt.% NaCl + 1.5 mol/L S_2_O_3_^2−^
*R*_S_ (Ωcm^−2^)	31.77	27.21	25.64	24.31
*Q*_f_ (Ω^−1^s^n^cm^−2^)	2.060 × 10^−3^	1.847 × 10^−2^	2.587 × 10^−2^	1.180 × 10^−4^
*n*_f_	0.3691	0.8341	0.8190	0.4635
*R*_f_ (Ωcm^−2^)	37.37	5.15	3.48	1.71
*Q*_dl_ (Ω^−1^s^n^cm^−2^)	1.085 × 10^−2^	4.269 × 10^−2^	1.802 × 10^−2^	4.651 × 10^−2^
*n*_dl_	0.5156	0.7140	0.8545	0.8000
*R*_ct_ (Ωcm^−2^)	217	539	472	457

**Table 5:** Optimal EEC parameters obtained from the dissolution of L245 in different corrosive media (15 d).

EEC Parameters	3.5 wt.% NaCl	3.5 wt.% NaCl + 0.5 mol/L S_2_O_3_^2−^	3.5 wt.% NaCl + 1.0 mol/L S_2_O_3_^2−^	3.5 wt.% NaCl + 1.5 mol/L S_2_O_3_^2−^
*R*_S_ (Ωcm^−2^)	6.240	0.690	0.549	2.599
*Q*_f_ (Ω^−1^s^n^cm^−2^)	1.420 × 10^−3^	1.966 × 10^−3^	7.457 × 10^−3^	2.022 × 10^−2^
*n*_f_	0.8490	0.8311	0.7952	0.7638
*R*_f_ (Ωcm^−2^)	41	110	61	22
*Q*_dl_ (Ω^−1^s^n^cm^−2^)	1.156 × 10^−2^	1.807 × 10^−2^	1.378 × 10^−2^	2.416 × 10^−2^
*n*_dl_	0.8289	0.5769	0.5503	0.7082
*R*_ct_ (Ωcm^−2^)	201.4	627	525	487

## Data Availability

The original contributions presented in this study are included in the article. Further inquiries can be directed to the corresponding authors.
